# Book Reviews

**Published:** 1832-02

**Authors:** 


### BIBLIOGRAPHICAL NOTICES.

*Researches to establish the Truth of the Linnæan Doctrine of Animate Contagions; wherein the Origin, Causes, Mode of Diffusion, and Cure of Epidemic Diseases, Spasmodic Cholera, Dysentery, Plague, Smallpox, Hooping Cough, Leprosy, &c. are illustrated by Facts, from the Natural History of Mankind, of Animals, and of Vegetables, and from the Phenomena of the Atmosphere.* By Adam Neale, m.d., Physician to his Majesty's Forces during the Peninsular War, and one of the Physicians Extraordinary to his late Royal Highness the Duke of Kent, &c.—8vo. pp. 258. Longman and Co. London.

Dr. Neale's researches have evidently cost him much pains, and his facts have been sought out of many sources; *but*, even allowing all his premises to be correct, they do not warrant the inferences he would draw therefrom. We wish we could impress upon the minds and memories of all speculative writers that the third term of a syllogism can never *con*clude more than the major proposition can fairly *in*clude; we should not then have so many sudden generalizations and visionary hypotheses, which shine but to dazzle, and light but to deceive.

The following is an extraordinary case; we extract it from a chapter headed "Curious Instances of Pestiferous Insects," and it is one of the strongest evidences offered by our author for the truth of the doctrine he advocates, viz.

that the diseases enumerated in his lengthened titlepage are produced by insects; i. e. that animalculæ are the causes of plague and pestilence as well as famine; but we need not point out to our intelligent readers the fallacy of the opinion, for, although disease and blight may be consantaneous, it does not follow that the one is the effect of the other: we should rather argue, that the same cause favors the production of both, that they are both the offspring of a common parent, or rather both the fosterchildren of a common nurse; for if accumulations of filth, of decaying animal and vegetable matter, &c. favor the developement of fevers, of cholera, and the whole host of ills that flesh is heir to, the very same circumstance will favor the generation of insects, who, thus nurtured, will increase and multiply to a most astonishing degree. But we have done: "Verbum non amplius" Neale.

"My readers will, I hope, permit me to suppress the name of the clergyman and the place where this event took place, as I am very sorry to say the reverend gentleman, who was much esteemed for his integrity, and well known by his literary genius, proceeded rather too far in the matter. The case was briefly this:

"In the month of July 17**, a very corpulent lady died at ***, in ****. Before her death she begged, as a particular favor, to be buried in the parochial church. She had died on the Wednesday, and on the following Saturday was buried according to her desire: the next day the clergyman preached her funeral sermon: the weather was uncommonly hot, and it ought to be observed, that for several months preceding her death a great drought had prevailed, not a drop of rain had fallen, and consequently it was an uncommonly sultry season. The succeeding Sunday, a week after the lady had been buried, the Protestant clergyman had a very full congregation, upwards of nine hundred persons attending, that being the day for administering the holy sacrament. The weather still continuing very hot, many were obliged, during the service, to walk out for a time, to avoid fainting away. It is the custom in Germany, that, when people wish to receive the sacrament, they neither eat nor drink, that day, till the ceremony is entirely over.

"The worthy clergyman preached about one hour and a quarter; he then consecrated the bread and wine, which ought to remain uncovered during the ceremony. There were about 180 communicants. A quarter of an hour after the ceremony, before they had quitted the church, more than sixty of them were taken ill; several died in the most violent agonies; others, of a more vigorous constitution, survived by the help of medical assistance. A most violent consternation prevailed through the whole congregation and town: it was concluded that the wine had been poisoned, and so it was generally believed. The sacristan and several others belonging to the vestry were immediately arrested, and put into irons.

"The clergyman, on the succeeding Sunday, preached with a deal of enthusiasm, and pointed out to his congregation several others concerned in the plot: this enthusiastic sermon was printed.

"The persons accused underwent very great hardships: during the space of a week they were confined in a dungeon, and some of them even put to the torture; but they still persisted in their innocence. On the Sunday following the magistrate ordered that a chalice of wine, uncovered, should be placed for the space of an hour upon the altar; which time had scarcely elapsed when they beheld the wine filled with myriads of insects, and, by tracing whence they came, it was at length perceived, by the rays of the sun, that they issued from the grave of the lady who had been buried the preceding fortnight. The people not belonging to the vestry were dismissed, and four men employed to open the grave and the coffin; in doing which, *two of them dropt down and expired upon the spot*, and the other two were only saved by the utmost exertion of medical talents. It is beyond the power of words to describe the horrid sight of the corpse when the coffin was opened: the whole was an entire mass of putrefaction, and it was now clearly demonstrated that the numerous insects, both large and small, together with the effluvia which issued from the body, had caused the pestilential infection which was a week before attributed to poison. On this discovery, the persons accused were, of course, instantly liberated, and every atonement made by the clergyman and magistrate for their misguided conduct."

---

*Parts I. II. III. The Principles and Practice of Obstetric Medicine, in a Series of Systematic Dissertations on Midwifery, and on the Diseases of Women and Children.* Illustrated by numerous Plates. By David D. Davis, M.D. M.R.S L., Professor of Midwifery in the University of London, &c.—4to. pp. 24. John Taylor, London.

We have no doubt that, when this work is completed, it will form a most instructive volume for the obstetric student and junior practitioner. The arrangement differs, and we think with advantage, from that which has usually been adopted by lecturers on the practice of midwifery, whose usual course has been to discuss the whole of the obstetric department of their subject at first, and then to take up in succession the diseases of women and children. The peculiar diseases of women are principally affections of organs which are proper to the sex: the whole of these organs will be described under the respective heads of Structure, Functions, and Morbid States of Structure and Functions. Thus, under one principal head, will be given every information concerning the pelvis; including its anatomy, its physiology, and its pathology. In the same manner will be treated all the organs and structures attached to or contained within the pelvis; first, anatomically in their healthy states; secondly, as agents in the performance of healthy functions; and thirdly, as the subjects of morbid conditions or actions. After having described the uterine system in its unimpregnated state, the modified anatomy, the altered or superadded functions, and peculiar diseases of the gravid uterus, will be treated of. A subsequent part of the work will contain a developement of those principles and rules of practice usually designated in books and in schools of medicine, *The Theory and Practice of Midwifery*. In the latter part of the work the Diseases of Children will be fully considered.

The work will be completed in between thirty and forty monthly parts; it will be illustrated by upwards of sixty plates, which will, as far as possible, accompany their respective letter-press, and illustrate generally the more practical and pathological subjects. The contents of the present and first parts of the work are of course entirely elementary; but, from the concise yet perspicuous manner in which the structure, dimensions, &c. of the female pelvis are described, we feel justified in forming a favorable opinion of the manner in which Dr. Davis will execute the subsequent parts.

---

*A Synopsis of the Bones, Ligaments, Muscles, Blood-vessels, and Nerves of the Human Body.* By William Sands Cox, Surgeon to the Dispensary, and Lecturer on Anatomy and Surgery at the School of Medicine in Birmingham. —8vo. pp. 310. Longman and Co., London.

The contents of this work need not be particularly detailed; they are expressed in the title. The merit of such a Synopsis must greatly depend upon the arrangement adopted, and that here followed appears to us to possess some advantage over similar productions, and to offer to the student, especially in the description of the bones, an easy plan, by which his memory will be much assisted in retaining the substance of the anatomical lectures he may attend.

We must be permitted to observe, that such a work should not have been "hastily arranged;" the author should have had no "fear that many errors will be found in the composition." The task of getting-up such a synopsis cannot be difficult; and, as it is easy, it ought not to have been performed with such haste as to render necessary the unsightly addition of a long list of errata.

